# Healthcare utilization and costs associated with *S. aureus* and *P. aeruginosa* pneumonia in the intensive care unit: a retrospective observational cohort study in a US claims database

**DOI:** 10.1186/s12913-015-0917-x

**Published:** 2015-06-21

**Authors:** Moe H. Kyaw, David M. Kern, Siting Zhou, Ozgur Tunceli, Hasan S. Jafri, Judith Falloon

**Affiliations:** MedImmune, Gaithersburg, USA; Current author address: Sanofi Pasteur, Swiftwater, PA USA; Healthcore, Wilmington, DE USA

**Keywords:** ICU, Pneumonia, Health economics, Resource utilization, *S. aureus*, *P. aeruginosa*

## Abstract

**Background:**

*Staphylococcus aureus* and *Pseudomonas aeruginosa* are major causes of pneumonia in intensive care unit (ICU) patients. Limited data exist regarding the health economic impact of *S. aureus* and *P. aeruginosa* pneumonias in the ICU setting.

**Methods:**

We conducted a retrospective observational cohort study using a 29.6 million enrollee US medical and pharmacy administrative claims database. ICU patients with *S. aureus* or *P. aeruginosa* infection per *International Classification of Diseases*, *9th ed.* coding between 01/01/2007-8/31/2012 were compared with ICU patients without any pneumonia or infections of interest. Primary outcomes were costs in 2012 US dollars, healthcare utilization and all-cause mortality associated with hospital-acquired *S. aureus* or *P. aeruginosa* pneumonia, and the relative odds of incurring higher costs due to a comorbid condition.

**Results:**

Patients with *S. aureus* or *P. aeruginosa* pneumonia had longer mean hospital (37.9 or 55.4 vs 7.2 days, *P* < .001) and ICU stays (6.9 or 14.8 vs 1.1 days, *P* < .001), a higher rate of mechanical ventilation (62.6 % or 62.3 % vs 7.4 %, *P* < .001), higher mortality (16.0 % or 20.2 % vs 3.1 %, *P* < .001), and higher total mean hospitalization costs ($146,978 or $213,104 vs $33,851, *P* < .001) vs controls. Pneumonia survivors had significantly increased risk of rehospitalization within 30 days (27.2 % or 31.1 % vs 15.3 %, *P* < .001). Comorbid conditions were not associated with increased cost in the pneumonia cohorts.

**Conclusions:**

Healthcare costs and resource utilization were high among ICU patients with *S. aureus* or *P. aeruginosa* pneumonia. Reducing the incidence of these infections could lead to substantial cost savings in the United States.

**Electronic supplementary material:**

The online version of this article (doi:10.1186/s12913-015-0917-x) contains supplementary material, which is available to authorized users.

## Background

*Staphylococcus aureus* (*S. aureus*) and *Pseudomonas aeruginosa* (*P. aeruginosa*) are responsible for much of the hospital-acquired pneumonia in the United States, accounting for approximately 28 %-47%and 18 % of hospitalizations, respectively [1, 2]. Despite preventive strategies, ventilator-associated pneumonia (VAP) remains the most frequent infection occurring in ICU patients [3]. *S. aureus* and *P. aeruginosa* cause 20 %-31 % and 21 %-24 % of VAP, [4–6] and 14 % and 13 % of non-VAP in intensive care unit (ICU) patients, respectively, in the United States [4]. These infections are associated with excess costs, lengths of stay, and mortality rates [2, 7–11]. Furthermore, infections due to antibiotic-resistant strains of *S. aureus* [6, 12, 13] and *P. aeruginosa* [14–18] add considerable disease burden and are associated with significantly higher costs, lengths of stay, and mortality versus infections caused by antibiotic-susceptible strains.

Limited data exist regarding the pharmacoeconomic and health outcomes of *S. aureus* and *P. aeruginosa* pneumonias in the ICU setting. This study assesses the impact of *S. aureus* or *P. aeruginosa* pneumonia in ICU patients on healthcare costs, utilization, and mortality both during hospitalization and subsequent to discharge from the hospital.

## Methods

### Design

This retrospective observational cohort study of administrative claims data from the HealthCore Integrated Research Environment for service dates from 01/01/2006 through 11/30/2012 was developed to assess costs and outcomes of *S. aureus* and *P. aeruginosa* pneumonia in ICU patients to guide the development of monoclonal antibodies designed to prevent these illnesses [19, 20]. The HealthCore database contains longitudinal claims data from one of the largest commercially insured population in the United States, including approximately 29.6 million enrollees in commercial health plans at the time of this study. On average, the population included in the HealthCore database has a higher household income, a lower proportion of individuals older than 65 years, and a higher proportion of whites compared to the overall US population. The observational and longitudinal aspects of the retrospective cohort design facilitated simultaneous capture of prior and follow-up data relative to the index event. The study was conducted in compliance with US federal regulations, the Health Insurance Portability and Accountability Act, and the Helsinki Declaration. Patient-specific data was de-identified, therefore, informed consent and institutional review board or ethics committee approval were not required for this study.

### Study population

**Fig. 1:**
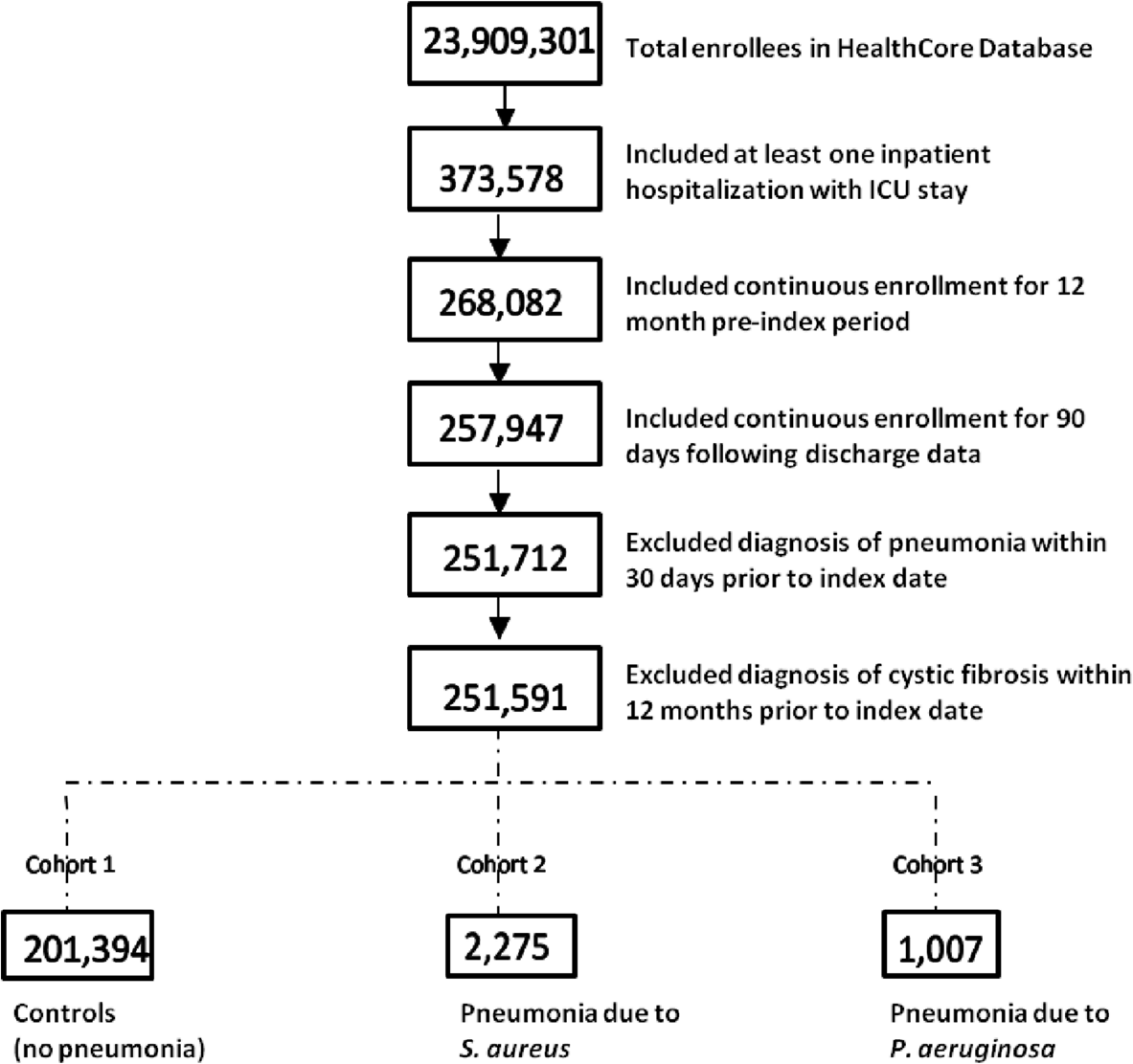
Inclusion/exclusion criteria, cohort definition, and patient counts. Cohort 1: Controls (no pneumonia); excluded the following conditions and Cohort 2 and Cohort 3: Viral pneumonia (ICD-9 dx 480.xx); Other pneumonia with specified bacteria: Pneumococcal pneumonia (481.xx), Other specified bacterial pneumonia (482.0x, 482.2x, 482.3x, 482.8x), Bronchopneumonia (485.xx); Pneumonia due to unspecified bacteria: Unspecified bacterial pneumonia (482.9x), Pneumonia, organism unspecified [bacterial or viral] (486.xx), Ventilator associated pneumonia (VAP) (997.31); Other pneumonias: Aspiration Pneumonia (507.0x), Post-procedural pneumonia (997.32); Septicemia due to staphylococcus or pseudomonas: Staphylococcal septicemia, unspecified (038.10), Methicillin susceptible Staphylococcus aureus septicemia (038.11), Methicillin resistant Staphylococcus aureus septicemia (038.12), Septicemia due to pseudomonas (038.43); Other bacterial infections due to staphylococcus or pseudomonas: Staphylococcus, unspecified (041.10), Methicillin susceptible Staphylococcus aureus (041.11), Methicillin resistant Staphylococcus aureus (041.12), Pseudomonas (041.7); Staphylococcal meningitis (320.3x); Bacteremia (790.7x). Cohort 2: Pneumonia due to staphylococcus (ICD-9 diagnosis: 482.4x). Cohort 3: Pneumonia due to pseudomonas (ICD-9 diagnosis: 482.1x). Cohort 2 and Cohort 3 are not necessarily mutually exclusive. Patients with diagnosis codes for both pneumonia due to staphylococcus and pneumonia due to pseudomonas during the index hospitalization visit are included in both cohorts (N = 132)

The eligible population comprised patients with ≥1 inpatient hospitalization that included an ICU stay and an admission and discharge between 01/01/2007 and 08/31/2012 (Fig. 1). Admissions to medical, surgical, cardiac, pediatric (including neonatal) ICUs were included. The first inpatient hospitalization that included any stay in an ICU was defined as the index hospitalization. To permit the identification of comorbid conditions and to assess long-term outcomes, eligible patients had ≥1 year of health plan enrollment before the admission date with continuous enrollment during the inpatient episode and ≥90 days of enrollment following the discharge date (unless death occurred first). Patients diagnosed with pneumonia within 30 days and patients diagnosed with cystic fibrosis within 12 months before the index period were excluded. The observation period for each patient was divided into pre-index, index, and 2 post-index time intervals. These were defined, respectively, as the 12 months before the index hospitalization, the index hospitalization itself, and both 30- and 90-day follow up periods post discharge from the index hospitalization.

Eligible ICU patients were divided into 3 primary groups based on *International Classification of Disease Clinical Modification, Ninth revision* (ICD-9) diagnosis codes: patients not diagnosed with pneumonia (Cohort 1–controls), patients with *S. aureus* pneumonia (Cohort 2), and patients with *P. aeruginosa* pneumonia (Cohort 3) (Fig. 1). Both primary and secondary diagnosis codes for the index hospitalizationwere used to assign subjects into groups.

### Outcomes

The main study outcomes included the total healthcare cost of the index hospitalization, the total length of stay, all-cause mortality during the index hospitalization, and the relative odds if the presence of a comorbid condition would result in higher total index hospitalization cost. The study also examined mechanical ventilation and ICU length of stay during the index hospitalization, and all-cause healthcare costs, all-cause mortality, hospital readmission, hospital and ICU lengths of stay, and antibiotic use during the post-index periods.

A comorbid condition was defined as the presence of ≥1 medical claim with a diagnosis code for the condition of interest for an individual during the 12 months prior to the index hospitalization. The total cost of care, including costs paid by the patient and those paid by the health plan, are included in all cost calculations. Post-index costs are reported as those incurred during inpatient hospitalizations (inpatient costs) as well as costs incurred in all places of service (total all-cause costs). Antibiotic use was captured via pharmacy and medical claims. The administrative claims data were linked to the Social Security Death Index to capture mortality during the post-index period.

### Analysis

Statistical analyses of the *S. aureus* and *P. aeruginosa* cohorts were performed separately against the control cohort. Cost outcomes were analyzed using a generalized linear model with gamma distribution and a log link function. Unadjusted differences in mean costs were reported for pre- and post-index periods, while means adjusted for covariates were reported for the primary outcome of index costs. Covariates for adjusted cost models included age (continuously), gender, health plan type, geographic region, Deyo-Charlson comorbidity index score (DCI), [21] and the analogous pre-index cost for index and post-index cost analyses only (index and post-index hospitalization cost models adjusted for pre-index hospitalization costs, total all-cause post-index costs adjusted for total pre-index costs).

Count variables and lengths of stay were analyzed using a generalized linear model with a negative binomial distribution with a log link function. Differences in index hospitalization length of stay were adjusted for the same covariates listed above, while unadjusted mean differences are reported for all pre- and post-index models. Logistic regression was used to analyze dichotomous variables with results reported as unadjusted odds ratios. Survival rates were calculated using Kaplan-Meier analysis, and unadjusted Cox-proportional hazards were used to analyze differences in survival between cohorts. The association between comorbid conditions and index hospitalization cost was analyzed separately within each cohort by dividing each cohort into quintiles of index hospitalization cost and calculating the prevalence of each comorbid condition within each quintile. Ordinal logistic regression was used to determine whether each comorbid condition was associated with higher cost quintiles, with results reported as unadjusted odds ratios.

For all variables, 95 % confidence intervals were presented. The magnitude of point estimates and the width of confidence intervals were used primarily to interpret results. Nominal *P*-values were calculated without adjustment for multiplicity; *P* < 0.05 was considered statistically significant.

## Results

In total, 251,591 patients were included in the final analysis. Figure 1 shows the inclusion/exclusion criteria, definitions, and sample size of patient cohorts. Baseline demographic characteristics of patients at the time of index hospitalization are summarized in Additional file 1: Table S1. Compared with controls, pneumonia patients were more likely to be older (mean age *S. aureus*: 60.5 years; *P. aeruginosa*: 62.8 years vs controls: 57.5 years) and male (*S. aureus*: 59.3 %; *P. aeruginosa*: 61.5 % vs controls: 56.3 %). Most patients in each cohort were covered by a preferred-provider type health plan (68 %-70 %).

### Costs

Table 1 presents a summary of the total and inpatient costs for the pre-index, index, and post-index (30 and 90 day) periods. Mean total costs for the index hospitalization were approximately 4 and 6 times greater in patients with *S. aureus* or *P. aeruginosa* pneumonia, respectively, than in those in the control group, resulting in incremental costs of > $100,000.

**Table 1:** Healthcare costs for ICU patients with *S. aureus* or *P. aeruginosa* pneumonia versus controls

				Pneumonia due to *S. aureus* vs No pneumonia		Pneumonia due to *P. aeruginosa* vs No pneumonia	
	No pneumonia, mean (SD) (n = 201,394)	*S. aureus* pneumonia, mean (SD) (n = 2275)	*P. aeruginosa* pneumonia, mean (SD) (n = 1007)	Difference^a^	*P* value^a^	Difference^b^	*P* value^b^
				(95 % CI)		(95 % CI)	
Pre-index costs,^c^ $ (12 months)							
Total all-cause costs	17,379 (38,923)	24,865 (55,676)	29,364 (65,771)	7,487 (6,024, 9,041)	<.001	11,985 (9,436, 14,776)	<.001
Inpatient hospitalization costs	5,985 (25,095)	10,389 (40,126)	11,885 (47,091)	4,405 (3,274, 5,674)	<.001	5,900 (4,015, 8,140)	<.001
Index hospitalization costs,^c^ $							
Total costs	33,851 (51,770)	146,978 (235,598)	213,104 (338,552)	100,216 (94,346, 106,360)	<.001	159,361 (146,758, 172,856)	<.001
Post-index costs,^c^ $ (30 days)							
Total all-cause costs	4,732 (18,044)	8,243 (23,838)	11,122 (42,503)	3,773 (3,236, 4,348)	<.001	6,327 (5,285, 7,484)	<.001
Inpatient hospitalization costs	2,610 (17,052)	4,836 (22,950)	7,447 (41,852)	2,710 (2,147, 3,343)	<.001	5,139 (3,924, 6,590)	<.001
Post-index costs,^c^ $ (90 days)							
Total all-cause costs	10,575 (27,653)	17,460 (39,286)	22,277 (54,113)	7,250 (6,231, 8,335)	<.001	10,842 (9,014, 12,852)	<.001
Inpatient hospitalization costs	4,913 (23,187)	9,281 (35,261)	13,527 (50,214)	4,731 (3,727, 5,860)	<.001	8118 (6,110, 10,513)	<.001
Index hospitalization + post-index costs (30 days),^c^ $							
Total all-cause costs	38,583 (57,402)	155,221 (240,527)	224,226 (346,331)	101,579 (95,681, 107,742)	<.001	162,756 (150,118, 176,254)	<.001
Inpatient hospitalization costs	36,461 (56,270)	151,813 (239,022)	220,552 (344,991)	102,287 (96,175, 108,687)	<.001	163,862 (150,714, 177,947)	<.001

*CI* confidence interval, *ICU* intensive care unit

^a^Differences in means are from gamma regression model (link = log). Statistical comparisons are comparing Cohort 3 (pneumonia due to *S. aureus*) with Cohort 1 (no pneumonia, reference group); ie, Difference = mean (Cohort 3) − mean (Cohort 1)

^b^Differences in means are from gamma regression model (link = log). Statistical comparisons are comparing Cohort 4 (pneumonia due to *P. aeruginosa*) with Cohort 1 (no pneumonia, reference group); ie, Difference = mean (Cohort 4) − mean (Cohort 1)

^c^Costs include $0 costs. All costs adjusted for calendar year, reported in 2012 dollars. All costs rounded to nearest dollar

All statistical models were controlled for the following variables: age (continuously), gender, health plan type, geographic region, and DCI comorbidity score. Models of index hospitalization costs and post-index costs were also controlled for the analogous healthcare costs during the 12 month pre-index period. Additional covariates were selected separately for each outcome using a forward selection method. The following additional covariates were considered for inclusion in the models: comorbid conditions (binary for each condition), prior healthcare utilization during the 12 month pre-index period (inpatient stays [0 vs 1+], emergency room visits [0 vs 1+], outpatient and office visits [continuous]), and prior antibiotic use during the 12 month pre-index period (0 vs. 1+)

During the first 30 days after index hospitalization discharge, patients in the *S. aureus* cohort incurred roughly 74 % higher mean costs than the control cohort ($8,243 vs $4732), and the *P. aeruginosa* cohort incurred more than double the mean cost compared with the control cohort. A similar pattern persisted during the 90 days following index hospitalization discharge.

During the index and 90-day post discharge periods inpatient costs are the major contributor for the overall costs in the *S. aureus* and *P. aeruginosa* cohorts and controls (>95 % vs 87.3 %, respectively). Inpatient and total costs were, respectively, $156,259 and $164,437 in the *S. aureus* pneumonia cohort and $226,631 and $235,381 in the *P. aeruginosa* pneumonia cohort compared with $38,765 and $44,427 for the control cohort (Table 1).

### Mortality within 90 days of discharge

Patients with pneumonia due to *S. aureus* or *P. aeruginosa* experienced significantly (P < .001) higher rates of mortality during the index hospitalization and the 90-day follow up period versus controls (Table 2). The rate of all-cause mortality from the index hospitalization through the end of the 90-day follow up period was also significantly higher (*P* < .001) in these cohorts. The mortality rates during the 90 days post-index period for patients who survived the index hospitalization were both significantly higher than the rate in controls (*P* < .001 for both). Overall survival rates during the post-index period (following index hospitalization discharge) were significantly lower (*P* < .001) in the *S. aureus* and *P. aeruginosa* cohorts compared with the control group.

**Table 2:** All-cause mortality of ICU patients with *S. aureus* or *P. aeruginosa* pneumonia versus controls

	Cohorts of interest			Comparisons			
				*S. aureus* pneumonia vs No pneumonia		*P. aeruginosa* pneumonia vs No pneumonia	
	No pneumonia (n = 201,394)	*S. aureus* pneumonia (n = 2275)	*P. aeruginosa* pneumonia (n = 1007)	Odds Ratio/Difference^a^ (95 % CI)	*P* Value^a^	Odds Ratio/Difference^b^ (95 % CI)	*P* Value^b^
Index hospitalization mortality							
No. (%) of patients	6,188 (3.1)	363 (16.0)	203 (20.2)	5.99 (5.34, 6.72)	<.001	7.97 (6.81, 9.31)	<.001
Post-index mortality							
No. (%) of patients	5,609 (2.8)	227 (10.0)	117 (11.6)	3.87 (3.37, 4.45)	<.001	4.59 (3.78, 5.57)	<.001
Mean (SD) time to death^c^	32.8 (27.1)	25.5 (25.8)	28.1 (28.4)	-7.30 (-10.51, -3.63)	<.001	-4.69 (-9.44, 1.02)	.10
Survival rate:				3.75 (3.28, 4.28)	<.001	4.40 (3.66, 5.28)	<.001
30 days (%)	98.5	93.4	92.9				
90 days (%)	97.2	90.0	88.4				
Index + post-index mortality							
No. (%) of patients	11,797 (5.9)	590 (25.9)	320 (31.8)	5.63 (5.11, 6.19)	<.001	7.49 (6.55, 8.56)	<.001
Mean (SD) time to death^c^	24.9 (28.3)	46.4 (47.6)	63.5 (59.3)	21.53 (17.39, 26.07)	<.001	38.57 (31.08, 47.07)	<.001

*CI* confidence interval, *ICU* intensive care unit

^a^Odds ratio (OR) from Chi-square test is used for categorical variables, negative binomial models are used to test for differences between means for count variables (number of events and length of stay). Statistical comparisons are comparing Cohort 3 (pneumonia due to *S. aureus*) with Cohort 1 (no pneumonia, reference group); ie, Difference = mean (Cohort 3) − mean (Cohort 1) and OR = Odds (Cohort 3)/Odds (Cohort 1)

^b^Odds ratio (OR) from Chi-square test is used for categorical variables, negative binomial models are used to test for differences between means for count variables (number of events and length of stay). Statistical comparisons are comparing Cohort 4 (pneumonia due to *P. aeruginosa*) with Cohort 1 (no pneumonia, reference group); i.e., Difference = mean (Cohort 4) − mean (Cohort 1) and OR = Odds (Cohort 4)/Odds (Cohort 1)

^c^Including only patients with mortality

### Healthcare utilization

Pre-index healthcare utilization in the *S. aureus* and *P. aeruginosa* cohorts was greater than in the control group. Patients who acquired these pneumonias during the index hospitalization had higher rates of inpatient hospitalizations and longer lengths of stay in the 12 months before the index hospitalization versus controls (Additional file 1: Table S3). The mean number of pre-index emergency room, office and outpatient visits was also generally higher among *S. aureus* and *P. aeruginosa* pneumonia patients than in controls (Additional file 1: Table S3). The percentage of patients with ≥1 visit to a skilled nursing facility in the pre-index period was much higher in both the *S. aureus* and *P. aeruginosa* cohorts than in the control cohort (*P* < .001). Antibiotic use was also significantly higher (Additional file 1: Table S3).

Healthcare utilization during the index hospitalization was higher among the *S. aureus* and *P. aeruginosa* cohorts versus the control cohort. Patients with *S. aureus* or *P. aeruginosa* pneumonia had significantly (*P* < .001) longer overall mean hospital stays (37.9 and 55.4 days, respectively) versus controls (7.2 days), ICU stays (6.9 and 14.8 days, respectively vs 1.1 days), and higher rates of mechanical ventilation during the index hospitalization (Table 3).

**Table 3:** Healthcare resource utilization for ICU patients with *S. aureus* or *P. aeruginosa* pneumonia versus controls during index hospitalization

	Cohorts of interest			*S. aureus* pneumonia vs No pneumonia		*P. aeruginosa* pneumonia vs No pneumonia	
	No pneumonia	*S. aureus* pneumonia	*P. aeruginosa* pneumonia	Odds Ratio/Difference^a^	*P* Value^a^	Odds Ratio/Difference^b^	*P* Value^b^
				(95 % CI)		(95 % CI)	
	N = 201,394	N = 2275	N = 1007				
Mean (SD) length of hospital stay per patient	7.2 (9.6)	37.9 (39.4)	55.4 (54.9)	30.69 (29.41, 32.00)	<.001	48.18 (45.43, 51.08)	<.001
Mean (SD) length of ICU/CCU stay per patient	1.1 (2.1)	6.9 (21.1)	14.8 (36.6)	5.75 (5.59, 5.91)	<.001	13.63 (13.21, 14.06)	<.001
No. (%) of patients with mechanical ventilation	14,960 (7.4)	1,425 (62.6)	627 (62.3)	20.89 (19.16, 22.78)	<.001	20.56 (18.08, 23.38)	<.001

*CI* confidence interval, *ICU* intensive care unit

^a^Odds ratio (OR) from Chi-square test is used for categorical variables, negative binomial models are used to test for differences between means for count variables (number of events and length of stay). Statistical comparisons are comparing Cohort 3 (pneumonia due to *S. aureus*) with Cohort 1 (no pneumonia, reference group); ie, Difference = mean (Cohort 3) − mean (Cohort 1) and OR = Odds (Cohort 3)/Odds (Cohort 1)

^b^Odds ratio (OR) from Chi-square test is used for categorical variables, negative binomial models are used to test for differences between means for count variables (number of events and length of stay). Statistical comparisons are comparing Cohort 4 (pneumonia due to *P. aeruginosa*) to Cohort 1 (no pneumonia, reference group); ie, Difference = mean (Cohort 4) – mean (Cohort 1) and OR = Odds (Cohort 4)/Odds (Cohort 1)

During the 30 day post discharge period, a significantly (*P* > .001) greater proportion of the survivors represented in the *S. aureus* and *P. aeruginosa* cohorts had inpatient hospitalizations and ICU visits) (Table 4) and skilled nursing facility visits (Additional file 1: Table S3) than did survivors in the control group, whereas the proportion of patients with office and outpatient visits were lower (*P* < .001) in the *S. aureus* and *P. aeruginosa* cohorts versus the control group (Additional file 1: Table S3). A similar pattern in healthcare utilization was seen within 90 days after index discharge.

**Table 4:** Healthcare resource utilization for ICU patients with *S. aureus* or *P. aeruginosa* pneumonia versus controls through 30 days post index hospitalization

	Cohorts of interest			*S. aureus* pneumonia vs No pneumonia		*P. aeruginosa* pneumonia vs No pneumonia	
	No pneumonia	*S. aureus* pneumonia	*P. aeruginosa* pneumonia	Odds Ratio/Difference^a^	*P* Value^a^	Odds Ratio/Difference^b^	*P* Value^b^
				(95 % CI)		(95 % CI)	
No. of patients completing 30 day follow up period^c^	n = 192,204	n = 1762	n = 732				
All-cause inpatient hospitalizations							
No. (%) of patients with ≥1 event	29,451 (15.3)	480 (27.2)	228 (31.1)	2.07 (1.86, 2.30)	<.001	2.50 (2.14, 2.93)	<.001
Mean (SD) events per patient with at least 1 event	1.3 (0.7)	1.6 (1.3)	1.5 (1.2)	0.30 (0.19, 0.42)	<.001	0.24 (0.09, 0.41)	.002
Mean (SD) length of stay per patient with at least 1 event	5.4 (5.5)	8.4 (7.3)	9.5 (7.7)	3.02 (2.38, 3.71)	<.001	4.15 (3.13, 5.29)	<.001
No. (%) of patients with ≥1 hospitalization for pneumonia due to *S. aureus*	52 (0.03)	46 (2.6)	13 (1.8)	99.06 (66.43, 147.71)	<.001	66.81 (36.22, 123.23)	<.001
No. (%) of patients with ≥1 hospitalization for pneumonia due to *P. aeruginosa*	31 (0.02)	16 (0.9)	38 (5.2)	56.81 (31.02, 104.05)	<.001	339.43 (210.00, 548.64)	<.001
ICU stays							
No. (%) of patients with ≥1 event	5027 (2.6)	84 (4.8)	55 (7.5)	1.86 (1.50, 2.33)	<.001	3.03 (2.30, 3.99)	<.001
Mean (SD) events per patient with at least 1 event	1.0 (0.2)	1.0 (0.1)	1.0 (0.0)	-0.01 (-0.21, 0.23)	.89	−0.03 (−0.26, 0.28)	.85
Mean (SD) length of stay per patient with at least 1 event	1.3 (1.6)	1.4 (3.0)	1.3 (1.2)	0.10 (-0.14, 0.40)	.44	0.02 (−0.27, 0.38)	.91
No. (%) of patients with ≥1 hospitalization for pneumonia due to *S. aureus*	34 (0.02)	18 (1.0)	7 (1.0)	58.34 (32.88, 103.49)	<.001	54.57 (24.11, 123.50)	<.001
No. (%) of patients with ≥1 hospitalization for pneumonia due to *P. aeruginosa*	18 (0.01)	8 (0.5)	17 (2.3)	48.72 (21.16, 112.18)	<.001	253.83 (130.29, 494.51)	<.001

*CI* confidence interval, *ICU* intensive care unit

^a^Odds ratio (OR) from Chi-square test is used for categorical variables, negative binomial models are used to test for differences between means for count variables (number of events and length of stay). Statistical comparisons are comparing Cohort 3 (pneumonia due to *S. aureus*) with Cohort 1 (no pneumonia, reference group); ie, Difference = mean (Cohort 3) − mean (Cohort 1) and OR = Odds (Cohort 3)/Odds (Cohort 1)

^b^Odds ratio (OR) from Chi-square test is used for categorical variables, negative binomial models are used to test for differences between means for count variables (number of events and length of stay). Statistical comparisons are comparing Cohort 4 (pneumonia due to *P. aeruginosa*) to Cohort 1 (no pneumonia, reference group); ie, Difference = mean (Cohort 4) – mean (Cohort 1) and OR = Odds (Cohort 4)/Odds (Cohort 1)

^c^Post-index utilization results include only patients who survived to the end of the 30 or 90 day post-discharge period

Survivors in the *S. aureus* and *P. aeruginosa* cohorts were more likely than controls to have a repeat pneumonia diagnosis during a subsequent hospital admission. Within the *S. aureus* cohort, during the 30-day and 90-day follow up period, respectively, re-hospitalization that included a diagnosis of *S. aureus* pneumonia accounted for 9.6 % and 10.0 % of inpatient admissions and 21.4 % and 15.3 % of readmissions to the ICUs. For the *P. aeruginosa* cohort, re-hospitalization that included a diagnosis of *P. aeruginosa* pneumonia accounted for 16.7 % and 20.0 % of inpatient admissions, and 30.9 % and 30.9 % of ICU admissions during these periods (Table 4, Table 5). Most readmissions due to pneumonia during the follow up period occurred during the first 30 days, particularly for the patients with *S. aureus* pneumonia.

**Table 5:** Healthcare resource utilization for ICU patients with *S. aureus* or *P. aeruginosa* pneumonia versus controls through 90 days post index hospitalization

	Cohorts of interest			*S. aureus* pneumonia vs No pneumonia		*P. aeruginosa* pneumonia vs No pneumonia	
	No pneumonia	*S. aureus* pneumonia	*P. aeruginosa* pneumonia	Odds Ratio/Difference^a^	*P* Value^a^	Odds Ratio/Difference^b^	*P* Value^b^
				(95 % CI)		(95 % CI)	
No. of patients completing 90 day follow up period^c^	n = 189,597	n = 1685	n = 687				
All-cause inpatient hospitalizations							
No. (%) of patients with ≥1 event	44,246 (23.3)	641 (38.0)	295 (42.9)	2.02 (1.83, 2.23)	<.001	2.48 (2.13, 2.88)	<.001
Mean (SD) events per patient with at least 1 event	1.5 (1.0)	1.9 (2.0)	2.0 (1.9)	0.45 (0.34, 0.56)	<.001	0.46 (0.31, 0.63)	<.001
Mean (SD) length of stay per patient with at least 1 event	7.8 (10.7)	14.7 (18.4)	17.5 (19.8)	6.96 (5.82, 8.19)	<.001	9.79 (7.85. 11.97)	<.001
No. (%) of patients with ≥1 hospitalization for pneumonia due to *S. aureus*	112 (0.06)	64 (3.8)	20 (2.9)	66.80 (48.95, 91.17)	<.001	50.74 (31.34, 82.14)	<.001
No. (%) of patients with ≥1 hospitalization for pneumonia due to *P. aeruginosa*	63 (0.03)	29 (1.7)	59 (8.6)	52.69 (33.85, 82.01)	<.001	282.61(196.46, 406.55)	<.001
ICU stays							
No. (%) of patients with ≥1 event	9,399 (5.0)	163 (9.7)	97 (14.1)	2.05 (1.75, 2.42)	<.001	3.15 (2.54, 3.91)	<.001
Mean (SD) events per patients with at least 1 event	1.1 (0.4)	1.1 (0.3)	1.2 (0.5)	-0.02 (-0.17, 0.15)	.80	0.10 (-0.10, 0.34)	.37
Mean (SD) length of stay per patients with at least 1 event	1.6 (3.6)	3.5 (10.1)	1.9 (3.6)	1.92 (1.46, 2.44)	<.001	0.34 (-0.01, 0.77)	.06
No. (%) of patients with ≥1 hospitalization for pneumonia due to *S. aureus*	66 (0.03)	25 (1.5)	9 (1.3)	43.25 (27.23, 68.70)	<.001	38.13 (18.93, 76.81)	<.001
No. (%) of patients with ≥1 hospitalization for pneumonia due to *P. aeruginosa*	32 (0.02)	17 (1.0)	30 (4.4)	60.38 (33.46, 108.94)	<.001	270.47 (163.40, 447.69)	<.001

*CI* confidence interval, *ICU* intensive care unit

^a^Odds ratio (OR) from Chi-square test is used for categorical variables, negative binomial models are used to test for differences between means for count variables (number of events and length of stay). Statistical comparisons are comparing Cohort 3 (pneumonia due to *S. aureus*) with Cohort 1 (no pneumonia, reference group); ie, Difference = mean (Cohort 3) − mean (Cohort 1) and OR = Odds (Cohort 3)/Odds (Cohort 1)

^b^Odds ratio (OR) from Chi-square test is used for categorical variables, negative binomial models are used to test for differences between means for count variables (number of events and length of stay). Statistical comparisons are comparing Cohort 4 (pneumonia due to *P. aeruginosa*) to Cohort 1 (no pneumonia, reference group); ie, Difference = mean (Cohort 4) – mean (Cohort 1) and OR = Odds (Cohort 4)/Odds (Cohort 1)

^c^Post-index utilization results include only patients who survived to the end of the 30 or 90 day post-discharge period

### Comorbidities

The most common prespecified comorbidities identified by coding during the 12-month pre-index period across the 3 cohorts were hypertension, diabetes, coronary heart disease, anemia, and chronic obstructive pulmonary disease (Additional file 1: Table S4). Almost all comorbidities were more prevalent in patients with *S. aureus* or *P. aeruginosa* pneumonia than in controls. The mean DCI score 2.3 for the *S. aureus* and 2.5 for the *P. aeruginosa* cohort; both were significantly higher than in the control cohort (mean DCI = 1.7).

Additional file 1: Table S5 displays the prevalence of comorbidities within the cost quintiles for each cohort. Most of the comorbid conditions were not associated with significantly higher costs within the *S. aureus* and *P. aeruginosa* cohorts during the index hospitalization (ie, the odds ratio of the likelihood of appearing in a high cost quintile in the presence of the comorbidity versus the absence of the comorbidity was not significantly greater than 1; Table 4). Hepatitis B infection and solid organ transplant were associated with higher costs within the *S. aureus* cohort only, but the *P* values were of marginal significance given the small sample sizes.

Nearly half of the comorbid conditions, including 4 cardiovascular conditions, were associated with significantly lower index hospitalization costs for either one or both of the *S. aureus* and *P. aeruginosa* cohorts (Table 6). Congestive heart failure, peripheral artery disease, and chronic obstructive pulmonary disease were associated with significantly lower costs in both the *S. aureus* and *P. aeruginosa* cohorts. Additionally, myocardial infarction, other coronary heart disease, and dementia were associated with lower costs in the *S. aureus* cohort; asthma, immunosuppression, human immunodeficiency virus infection, and Hepatitis B infection were associated with lower costs in the *P. aeruginosa* cohort. However, the results for immunosuppression, human immunodeficiency virus, and hepatitis B should be interpreted with caution due to the small sample sizes (Additional file 1: Table S4).

**Table 6:** Impact of comorbidities on index hospitalization costs for ICU patients with *S. aureus* or *P. aeruginosa* pneumonia

Comorbidity	*S. aureus* pneumonia		*P. aeruginosa* pneumonia	
	Odds Ratio^a^	*P* Value^a^	Odds Ratio^a^	*P* Value^a^
	(95 % CI)		(95 % CI)	
Diabetes	1.10 (0.93, 1.31)	.27	1.12 (0.86, 1.46)	.40
Myocardial infarction	0.58 (0.42, 0.82)	.002	1.14 (0.71, 1.82)	.59
Congestive heart failure	0.74 (0.60, 0.92)	.007	0.72 (0.52, 0.99)	.04
Peripheral artery disease	0.69 (0.54, 0.88)	.003	0.70 (0.49, 0.99)	.04
Stroke, TIA, cerebrovascular disease	0.81 (0.65, 1.01)	.06	1.17 (0.83, 1.65)	.37
Hypertension	0.96 (0.80, 1.14)	.62	1.16 (0.88, 1.52)	.29
Other coronary heart disease	0.80 (0.66, 0.97)	.03	0.94 (0.71, 1.24)	.65
Anemia	0.88 (0.72, 1.07)	.20	1.12 (0.84, 1.50)	.44
COPD	0.68 (0.57, 0.82)	<.001	0.46 (0.35, 0.60)	<.001
Asthma	0.95 (0.76, 1.20)	.67	0.60 (0.43, 0.84)	.003
Renal disease	0.90 (0.70, 1.15)	.38	1.06 (0.73, 1.52)	.77
Chronic liver disease	1.17 (0.87, 1.58)	.29	0.85 (0.54, 1.33)	.47
Neutropenia	1.53 (0.91, 2.58)	.11	0.59 (0.29, 1.19)	.14
Immunosuppression	1.05 (0.51, 2.16)	.89	0.39 (0.16, 0.96)	.04
HIV	1.00 (0.34, 2.90)	.996	0.27 (0.08, 0.88)	.03
Hepatitis B	4.77 (1.05, 21.72)	.04	0.04 (0.00, 0.74)	.03
Hepatitis C	0.96 (0.45, 2.05)	.92	0.57 (0.19, 1.70)	.31
Obesity/Overweight	1.03 (0.78, 1.36)	.86	1.15 (0.76, 1.75)	.50
Dementia	0.55 (0.35, 0.86)	.009	1.76 (0.75, 4.15)	.20
Dialysis	0.62 (0.33, 1.15)	.13	1.21 (0.53, 2.78)	.65
Leukemia	0.61 (0.27, 1.35)	.22	1.03 (0.43, 2.47)	.95
Lymphoma	1.60 (0.91, 2.82)	.11	1.21 (0.56, 2.58)	.63
Cancer other than leukemia or lymphoma	0.88 (0.58, 1.34)	.56	0.93 (0.51, 1.70)	.82
Bone marrow transplant	0.89 (0.15, 5.30)	.89	0.61 (0.11, 3.43)	.58
Solid organ transplant	1.90 (1.02, 3.53)	.04	1.31 (0.66, 2.57)	.44

*CI* confidence interval, *COPD* chronic obstructive pulmonary disease, *HIV* human immunodeficiency virus, *ICU* intensive care unit, *TIA* transient ischemic attack

^a^Ordinal logistic regression odds ratio (OR) is used to test the association between the presence of each comorbidity and index hospitalization cost quintiles, ie, OR = Odds (patient with comorbidity in high cost quintile)/ Odds (patient without comorbidity in high cost quintile). OR >1 means presence of the comorbidity leads to greater costs, whereas OR <1 means presence of the comorbidity leads to lower costs

All statistical models were controlled for the following variables: age (continuously), gender, health plan type, geographic region, prior inpatient hospitalization costs during the 12 month pre-index period, prior healthcare utilization during the 12 month pre-index period (inpatient stays [0 vs 1+], emergency room visits [0 vs 1+], outpatient and office visits [continuous]), and prior antibiotic use during the 12 month pre-index period (0 vs. 1+)

An exploratory analysis of congestive heart failure was performed that stratified patients by age and mortality (Additional file 1: Table S6) to control for potential correlations between comorbidities and patient characteristics and outcomes (Medicare eligibility and death) that may result in lower hospitalization costs. The results showed odds ratios <1 despite these additional adjustments in the models. Similar results were found in exploratory analyses of other pre-specified conditions with odds ratios <1.

## Discussion

This is the largest US claims database study of healthcare costs and outcomes for ICU patients with a diagnosis of *S. aureus* or *P. aeruginosa* pneumonia. Our findings highlight the comprehensive economic consequences attributed to *S. aureus* and *P. aeruginosa* pneumonia and can permit policy makers, payers, and healthcare providers to assess the effect of prevention or therapeutic efforts on the cost and morbidity of these ICU infections.

In our study, ICU patients with pneumonia had substantially higher healthcare costs during the index admission: > $213,000 for *P. aeruginosa* pneumonia and > $146,000 for with *S. aureus* pneumonia versus >$33,000 for patients without pneumonia. Increased utilization continued after index hospitalization discharge, with total healthcare costs through 90 days post discharge of > $17,000 for patients with *S. aureus* pneumonia and > $22,000 for patients with *P. aeruginosa* pneumonia versus > $10,000 for patients without pneumonia. Patients with *S. aureus* or *P. aeruginosa* pneumonia had estimated incremental index hospitalization costs of $100,000-$160,000 and total healthcare costs of $107,000-$167,000 versus ICU patients without pneumonia. In previous studies of the general US inpatient population, the mean cost of hospital care in patients with hospital-acquired pneumonia was $72,000 versus $46,400 to $65,292 for patients without pneumonia [2, 22]. A German study reported an excess mean cost of DM29,610 (equivalent to $16,824 in 2001) for hospital-acquired pneumonia in ICU patients compared with ICU patients without pneumonia [23]. Previous US studies in VAP patients estimated that the incremental costs were $39,000 to $100,000 for VAP patients versus ICU patients without VAP, which is consistent with our observations [24, 25].

Our findings showed that *S. aureus* pneumonia and, especially, *P. aeruginosa* pneumonia had prolonged hospitalizations (>48 days longer for *P. aeruginosa* and >30 days longer for *S. aureus*) and ICU stays (approximately 15 days for those with *P. aeruginosa* pneumonia, 7 days for those with *S. aureus* pneumonia, compared with 1 day for those without pneumonia). Mortality was also substantially higher: >20 % of those with *P. aeruginosa* pneumonia and 16 % of those with *S. aureus* pneumonia died during the index hospitalization versus approximately 3 % of control patients, and this trend continued after discharge. The findings of longer ICU lengths of stay and longer hospital lengths of stay are consistent with prior studies [2, 22–24].

We found significantly higher rehospitalization rates in patients with *S. aureus* and *P. aeruginosa* pneumonia versus controls. Readmission after discharge can be costly and problematic for the patient, hospitals, and payers. It was estimated that hospital readmissions cost Medicare $17.5 billion alone annually in the United States [26]. Financial penalties are now incurred for hospitals with excess 30-day readmission in the United States. Therefore, preventing *S. aureus* and *P. aeruginosa* pneumonias could reduce the substantial costs of and healthcare utilization associated with rehospitalization in patients with these infections, resulting in benefits for patients, payers, and healthcare providers.

ICU patients with *S. aureus* or *P. aeruginosa* pneumonia were older, more likely to be male, and in poorer general health than those who did not have pneumonia. Most patients (>62 %) were mechanically ventilated; this is likely to be an underestimate, because mechanical ventilation is not uniformly recorded in a claims database. These mechanically-ventilated patients represent both those with VAP and those with non-ventilator-associated pneumonia that required ventilator support. The demographic skew to older, male patients has been previously reported in patients with VAP; however, in some studies, patients with VAP were younger than were those without VAP, which may reflect the contribution of trauma patients to the demographics [24, 27].

The findings regarding the impact of comorbid conditions on the hospitalization costs of *S. aureus* or *P. aeruginosa* pneumonia are unique to the present study. The results indicate that while the presence of comorbid conditions increases the likelihood that an ICU patient will develop *S. aureus* or *P. aeruginosa* pneumonia, the presence of comorbidities does not inflate the costs of hospital care for the patient. A number of comorbid conditions were associated with lower, rather than higher, hospitalization costs for *S. aureus* or *P. aeruginosa* pneumonia. Exploratory analysis demonstrated that the lower costs are not solely due to higher mortality or higher rates of Medicare coverage among patients with the comorbid conditions. It is unknown why these inverse associations exist.

Claims data represent an excellent starting point for the examination of health outcomes, treatment patterns, healthcare resource utilization, and costs. However, our analyses have several limitations. This study was retrospective and used ICD-9 codes to include and exclude subjects from specific cohorts, a method associated with known limitations [28]. Because the included codes were selected to be specific rather than sensitive, the numbers of patients identified cannot be used to establish incidence rates. It is possible that other pneumonia codes, including the code for VAP, included some episodes of *P. aeruginosa* or *S. aureus* pneumonia. Comorbidities were identified by their presence in coded diagnoses in the 12 months before the index hospitalization; this approach is likely very sensitive and intended to capture as many comorbid conditions as possible but likely has limited specificity due to the criteria of only requiring one diagnosis code. Diagnoses made during the index hospitalization were not included, because the goal of the current analysis was to assess the information available to clinicians upon admission; this may have contributed to underdiagnosis of co-morbid conditions. Future studies may want to examine the association between costs and both pre-existing and new comorbidities to explore the impact of how comorbidities are defined. Additionally, separating patients into those hospitalized for a life-threatening pneumonia from those who acquired pneumonia in the hospital would be useful in assessing prevention strategies, however claims database analyses do not permit separation into these groups. Furthermore, the database only included patients who had commercial healthcare insurance and may not be representative of the overall US population; patients covered solely by Medicare or Medicaid were excluded. However, the claims database comprised patients aged ≥18 years in a managed care setting and included substantial numbers of subjects of Medicare age; moreover, results may be generalizable to similar patient groups. Despite these limitations, our results provide accurate assessments of resource utilizations and costs in these patients due to appropriate adjustments in the statistical models.

## Conclusion

Overall, these results indicate that *S. aureus* and *P. aeruginosa* pneumonia in ICU patients impose substantial costs, healthcare utilization, and burden for patients [29]. The demand for critical care services is increasing as the aging US population requires a greater level of critical and end of life care, and emergency departments are accelerating their rates of ICU admissions [30]. These services are costly; in 2005, ICUs in the United States accounted for 23.2 million patient days and $81.7 billion in expenditures. Our results highlight the economic importance of effective interventions to reduce the burden of *S. aureus* and *P. aeruginosa* pneumonia in ICUs in the United States.

## Additional file

Additional file 1: Table S1. Baseline demographic characteristics of ICU patients with *S. aureus* or *P. aeruginosa* pneumonia versus controls. **Table S2.** Non-hospital medical and pharmacy costs for ICU patients with *S. aureus* or *P. aeruginosa* pneumonia versus controls. **Table S3.** Pre- and post-index healthcare resource utilization for ICU patients with *S. aureus* or *P. aeruginosa* pneumonia versus controls. **Table S4.** Comorbidities of ICU patients with *S. aureus* and *P. aeruginosa* pneumonia versus controls. **Table S5.** Prevalence of comorbid conditions by quintile of index hospitalization cost for ICU patients with *S. aureus* or *P. aeruginosa* pneumonia versus controls. **Table S6.** Exploratory analysis of the impact of congestive heart failure on index hospitalization costs in ICU patients with hospital acquired pneumonia due to *S. aureus.* **Table S7.** Healthcare resource utilization for ICU patients with *S. aureus* or *P. aeruginosa* pneumonia versus controls pre-index hospitalization (12 months).
