# Supplementary material for: Healthcare utilization and costs associated with S. aureus and P. aeruginosa pneumonia in the intensive care unit: a retrospective observational cohort study in a US claims database
Source: BMC Health Serv Res. 2015 Jun 21;15:241. doi: 10.1186/s12913-015-0917-x (PMC4475310; doi:10.1186/s12913-015-0917-x)
Supplement: Additional file 1: Table S1. — Baseline demographic characteristics of ICU patients with S. aureus or P. aeruginosa pneumonia versus controls. Table S2. Non-hospital medical and pharmacy costs for ICU patients with S. aureus or P. aeruginosa pneumonia versus controls. Table S3. Pre- and post-index healthcare resource utilization for ICU patients with S. aureus or P. aeruginosa pneumonia versus controls. Table S4. Comorbidities of ICU patients with S. aureus and P. aeruginosa pneumonia versus controls. Table S5. Prevalence of comorbid conditions by quintile of index hospitalization cost for ICU patients with S. aureus or P. aeruginosa pneumonia versus controls. Table S6. Exploratory analysis of the impact of congestive heart failure on index hospitalization costs in ICU patients with hospital acquired pneumonia due to S. aureus. Table S7. Healthcare resource utilization for ICU patients with S. aureus or P. aeruginosa pneumonia versus controls pre-index hospitalization (12 months). [file 12913_2015_917_MOESM1_ESM.docx]

Additional Files

This supplementary material has been provided by the authors to give readers additional information about their work.

Additional File 1

Format – Word, DOCX

Title of Data - Supplementary Tables

eTable 1. Baseline demographic characteristics of ICU patients with *S. aureus* or *P. aeruginosa* pneumonia versus controls

|  | **Cohorts of interest** | | | | **Comparisons** | | | | | |
| --- | --- | --- | --- | --- | --- | --- | --- | --- | --- | --- |
|  | **No pneumonia (n = 201,394)** | ***S. aureus* pneumonia (n = 2,275)** | ***P. aeruginosa* pneumonia (n = 1,007)** | | ***S. aureus* pneumonia vs No pneumonia** | | | ***P. aeruginosa* pneumonia vs No pneumonia** | | |
|  |  |  |  |  | **Odds Ratio / Difference^a^ (95% CI)** | ***P* Value^a^** | | **Odds Ratio / Difference^b^ (95% CI)** | | ***P* Value^b^** |
| **No. (%) by age, y** | | | | | | | | | | |
| 0–17 | 10,509 (5.2) | 59 (2.6) | 19 (1.9) | | 0.48 (0.37, 0.63) | <.001 | | 0.35 (0.22, 0.55) | | <.001 |
| 18–49 | 45,910 (22.8) | 486 (21.4) | 167 (16.6) | | 0.92 (0.83, 1.02) | .11 | | 0.67 (0.57, 0.80) | | <.001 |
| 50–64 | 73,209 (36.4) | 767 (33.7) | 334 (33.2) | | 0.89 (0.82, 0.97) | .009 | | 0.87 (0.76, 0.99) | | .04 |
| 65–74 | 30,611 (15.2) | 374 (16.4) | 199 (19.8) | | 1.10 (0.98, 1.23) | .10 | | 1.38 (1.18, 1.61) | | <.001 |
| 75+ | 41,155 (20.4) | 589 (25.9) | 288 (28.6) | | 1.36 (1.24, 1.50) | <.001 | | 1.56 (1.36, 1.79) | | <.001 |
| Mean (SD) age | 57.5 (19.6) | 60.5 (18.9) | 62.8 (17.2) | | 2.99 (2.18, 3.80) | <.001 | | 5.28 (4.07, 6.50) | | <.001 |
| **No. (%) by gender** | | | | | | | | | | |
| Male | 113,352 (56.3) | 1,348 (59.3) | 619 (61.5) | | 1.13 (1.04, 1.23) | .005 | 1.24 (1.09, 1.41) | | <.001 | |
| Female | 88,042 (43.7) | 927 (40.7) | 388 (38.5) | | 0.89 (0.82, 0.96) | .005 | 0.81 (0.71, 0.92) | | <.001 | |
| **No. (%) by year of index hospitalization** | | | | | | | | | | |
| 2007 | 41,582 (20.6) | 507 (22.3) | 194 (19.3) | 1.10 (1.00, 1.22) | | .05 | 0.92 (0.79, 1.08) | | .28 | |
| 2008 | 38,563 (19.1) | 454 (20.0) | 193 (19.2) | 1.05 (0.95, 1.17) | | .33 | 1.00 (0.85, 1.17) | | .99 | |
| 2009 | 37,021 (18.4) | 420 (18.5) | 213 (21.2) | 1.01 (0.90, 1.12) | | .92 | 1.19 (1.02, 1.39) | | .02 | |
| 2010 | 34,631 (17.2) | 399 (17.5) | 208 (20.7) | 1.02 (0.92, 1.14) | | .67 | 1.25 (1.08, 1.46) | | .004 | |
| 2011 | 31,383 (15.6) | 353 (15.5) | 145 (14.4) | 1.00 (0.89, 1.12) | | .93 | 0.91 (0.76, 1.09) | | .30 | |
| 2012 | 18,214 (9.0) | 142 (6.2) | 54 (5.4) | 0.67 (0.57, 0.80) | | <.001 | 0.57 (0.43, 0.75) | | <.001 | |
| **No. (%) by health plan type** | | | | | | | | | | |
| HMO | 53,280 (26.5) | 622 (27.3) | 286 (28.4) | | 1.05 (0.95, 1.15) | .34 | 1.11 (0.96, 1.27) | | .16 | |
| PPO | 139,226 (69.1) | 1,581 (69.5) | 693 (68.8) | | 1.02 (0.93, 1.11) | .71 | 0.99 (0.86, 1.13) | | .83 | |
| CDHP | 8,888 (4.4) | 72 (3.2) | 28 (2.8) | | 0.71 (0.56, 0.90) | .004 | 0.62 (0.43, 0.90) | | .01 | |
| **No. (%) by geographic region** | | | | | | | | | | |
| Northeast | 35,404 (17.6) | 384 (16.9) | 173 (17.2) | | 0.95 (0.85, 1.06) | .38 | 0.97 (0.83, 1.15) | | .74 | |
| Midwest | 75,625 (37.6) | 855 (37.6) | 377 (37.4) | | 1.00 (0.92, 1.09) | .98 | 1.00 (0.88, 1.13) | | .94 | |
| South | 56,751 (28.2) | 614 (27.0) | 262 (26.0) | | 0.94 (0.86, 1.04) | .21 | 0.90 (0.78, 1.03) | | .13 | |
| West | 33,614 (16.7) | 422 (18.5) | 195 (19.4) | | 1.14 (1.02, 1.26) | .02 | 1.20 (1.03, 1.40) | | .02 | |

CDHP=Consumer Directed Health Plan; CI=confidence interval; HMO=Health Maintenance Organization; ICU=intensive care unit; PPO=Preferred Provider Organization; SD=standard deviation.

^a^Chi-square test odds ratio (OR) is used for categorical variables. Statistical comparisons are comparing Cohort 3 (pneumonia due to *S. aureus*) with Cohort 1 (no pneumonia, reference group); ie, OR = Odds (Cohort 3)/Odds (Cohort 1).

^b^Chi-square test odds ratio (OR) is used for categorical variables. Statistical comparisons are comparing Cohort 4 (pneumonia due to *P. aeruginosa*) with Cohort 1 (no pneumonia, reference group); ie, OR = Odds (Cohort 4)/Odds (Cohort 1).

eTable 2. Non-hospital medical and pharmacy costs for ICU patients with *S. aureus* or *P. aeruginosa* pneumonia versus controls

|  | **Cohorts of Interest** | | | | | | **Comparisons** | | | |
| --- | --- | --- | --- | --- | --- | --- | --- | --- | --- | --- |
|  | **No pneumonia, mean (SD)  (n = 201,394)** | | | ***S. aureus* pneumonia, mean (SD)  (n = 2,275)** | | ***P. aeruginosa* pneumonia, mean (SD)  (n = 1,007)** | ***S. aureus* pneumonia vs No pneumonia** |  | ***P. aeruginosa* pneumonia vs No pneumonia** |  |
|  |  |  |  |  |  |  | **Difference^a^**  **(95% CI)** | ***P* value^a^** | **Difference^b^**  **(95% CI)** | ***P* value^b^** |
| **Pre-index costs (12 months)** | | | | | | | | | | |
| All-cause Medical costs | $14,703  ($37,233) | | $20,881  ($53,490) | | $25,252  ($63,879) | | $6,177  ($4,851, $7,593) | <.001 | $10,549  ($8,186, $13,156) | <.001 |
| Inpatient hospitalization | $5,985  ($25,095) | | $10,389  ($40,126) | | $11,885  ($47,091) | | $4,405  ($3,274, $5,674) | <.001 | $5,900  ($4,015, $8,140) | <.001 |
| Emergency Department | $550  ($2,106) | | $669  ($3,271) | | $492  ($1,715) | | $119  ($55, $190) | <.001 | −$58  (−$127, $22) | .15 |
| Office visit | $1,525  ($5,310) | | $1,697  ($5,920) | | $2,443  ($12,962) | | $172  ($77, $272) | <.001 | $918  ($717, $1,137) | <.001 |
| Outpatient visit | $6,456  ($18,764) | | $7,394  ($23,990) | | $9,873  ($30,104) | | $938  ($420, $1,495) | <.001 | $3,417  ($2,400, $4,552) | <.001 |
| Other medical | $187  ($1,890) | | $732  ($3,676) | | $559  ($3,301) | | $544  ($472, $625) | <.001 | $372  ($291, $466) | <.001 |
| All-cause Pharmacy costs | $2,676  ($5,669) | | $3,984  ($7,827) | | $4,112  ($6,948) | | $1,308  ($1,027, $1,611) | <.001 | $1,436  ($1,009, $1,913) | <.001 |
| **Post-index costs (30 days)** | | | | | | | | | | |
| All-cause Medical costs | $4,391  ($17,941) | | $7,875  ($23,793) | | $10,784  ($42,490) | | $3,793  ($3,224, $4,407) | <.001 | $6,416  ($5,295, $7,674) | <.001 |
| Inpatient hospitalization | $2,610  ($17,052) | | $4,836  ($22,950) | | $7,447  ($41,852) | | $2,710  ($2,147, $3,343) | <.001 | $5,139  ($3,924, $6,590) | <.001 |
| Emergency Department | $110  ($671) | | $112  ($578) | | $118  ($618) | | $13  ($3, $24) | .01 | $22  ($6, $41) | .006 |
| Office visit | $242  ($929) | | $235  ($849) | | $203  ($766) | | −$14  (−$27, −$1) | .03 | $22  ($6, $41) | .006 |
| Outpatient visit | $1,095  ($4,275) | | $1,524  ($4,548) | | $1,632  ($5,295) | | $366  ($265, $477) | <.001 | $319  ($175, $483) | <.001 |
| Other medical | $334  ($1,543) | | $1,168  ($3,625) | | $1,383  ($4,456) | | $1,203  ($1,072, $1,346) | <.001 | $1,213  ($1,021, $1,434) | <.001 |
| All-cause Pharmacy costs | $341  ($930) | | $368  ($926) | | $338  ($896) | | −$21  (−$38, −$3) | .03 | −$42  (−$65, −$16) | .002 |
|  |  | |  | |  | |  |  |  |  |
| **Post-index costs (90 days)** | | | | | | | | | | |
| All-cause Medical costs | $9,634  ($27,193) | | $16,472  ($38,867) | | $21,395  ($53,857) | | $7,292  ($6,237, $8,423) | <.001 | $11,054  ($9,129, $13,189) | <.001 |
| Inpatient hospitalization | $4,914  ($23,187) | | $9,281  ($35,261) | | $13,527  ($50,214) | | $4,731  ($3,727, $5,860) | <.001 | $8,118  ($6,110, $10,513) | <.001 |
| Emergency Department | $225  ($1,043) | | $252  ($967) | | $268  ($1,039) | | $41  ($19, $66) | <.001 | $81  ($43, $125) | <.001 |
| Office visit | $646  ($2,462) | | $661  ($2,771) | | $515  ($1,353) | | $8  (−$23, $41) | .62 | −$126  (−$161, −$89) | <.001 |
| Outpatient visit | $3,281  ($10,323) | | $3,881  ($9,731) | | $4,357  ($11,455) | | $622  ($384, $879) | <.001 | $611  ($260, $1,005) | <.001 |
| Other medical | $567  ($2,645) | | $2,397  ($7,774) | | $2,729  ($8,937) | | $3,096  ($2,768, $3,457) | <.001 | $2,662  ($2,247, $3,145) | <.001 |
| All-cause Pharmacy costs | $942  ($2,066) | | $988  ($2,356) | | $882  ($2,046) | | −$84  (−$126, −$39) | <.001 | −$135  (−$193, −$72) | <.001 |
| **Index hospitalization + post-index costs (30 days)** | | | | | | | | | | |
| Total costs | $38,583  ($57,402) | | $155,221  ($240,527) | | $224,226  ($346,331) | | $101,579  ($95,681, $107,742) | <.001 | $162,756  ($150,118, $176,254) | <.001 |
| Inpatient costs | $36,461  ($56,270) | | $151,813  ($239,022) | | $220,552  ($344,991) | | $102,287  ($96,175, $108,687) | <.001 | $163,862  ($150,714, $177,947) | <.001 |
| **Index hospitalization + post-index costs (90 days)** | | | | | | | | | | |
| Total costs | | $44,427  ($63,367) | $164,437  ($247,800) | | $235,381  ($351,603) | | $105,044  ($98,916, $111,440) | <.001 | $166,988  ($154,054, $180,780) | <.001 |
| Inpatient costs | | $38,765  ($59,548) | $156,259  ($243,997) | | $226,631  ($348,188) | | $103,586  ($97,305, $110,164) | <.001 | $166,545  ($153,045, $181,010) | <.001 |

CI=confidence interval; ICU=intensive care unit; SD=standard deviation.

^a^Differences in means are from gamma regression model (link=log). Statistical comparisons are comparing Cohort 3 (pneumonia due to *S. aureus*) with Cohort 1 (no pneumonia, reference group); ie, Difference = mean (Cohort 3) – mean (Cohort 1).

^b^Differences in means are from gamma regression model (link=log). Statistical comparisons are comparing Cohort 4 (pneumonia due to *P. aeruginosa*) with Cohort 1 (no pneumonia, reference group); ie, Difference = mean (Cohort 4) – mean (Cohort 1).

^c^Costs include $0 costs. All costs adjusted for calendar year, reported in 2012 dollars. All costs rounded to nearest dollar.

All statistical models were controlled for the following variables: age (continuously), gender, health plan type, geographic region, and DCI comorbidity score. Models of post-index costs were also controlled for the analogous healthcare costs during the 12 month pre-index period. Additional covariates were selected separately for each outcome using a forward selection method. The following additional covariates were considered for inclusion in the models: comorbid conditions (binary for each condition), prior healthcare utilization during the 12 month pre-index period (inpatient stays [0 vs 1+], emergency room visits [0 vs 1+], outpatient and office visits [continuous]), and prior antibiotic use during the 12 month pre-index period (0 vs 1+).

**eTable 3. Pre- and post-index healthcare resource utilization for ICU patients with *S. aureus* or *P. aeruginosa* pneumonia versus controls**

|  | **Cohorts of Interest** | | | | | | | | | **Comparisons** | | | | | | | | | | | | |
| --- | --- | --- | --- | --- | --- | --- | --- | --- | --- | --- | --- | --- | --- | --- | --- | --- | --- | --- | --- | --- | --- | --- |
|  | **No pneumonia, mean (SD) (n = 201,394)** | ***S. aureus* pneumonia, mean (SD) (n = 2,275)** | | ***P. aeruginosa* pneumonia, mean (SD) (n = 1,007)** | | | | | | ***S. aureus* pneumonia vs No pneumonia** | | | | | | | ***P. aeruginosa* pneumonia vs No pneumonia** | | | | | |
|  |  |  |  |  |  |  |  |  |  | **Difference^a^ (95% CI)** | | ***P* value^a^** | | | | | **Difference^b^ (95% CI)** | | | ***P* value^b^** | | |
| **Pre-index utilization (12 months)** | | | | | | | | | | | | | | | | | | | | | | |
| No. of patients | n = 201,394 | | n = 2,275 | n = 1,007 | | | | | |  | |  | | | | |  | | |  | | |
| **All-cause ED visits** | | | | | | | | | | | | | | | | | | | | | | |
| No. (%) of patients with ≥1 event | 53,684 (26.7) | | 687 (30.2) | | 276 (27.4) | | | | | 1.19 (1.09, 1.30) | | <.001 | | | 1.04 (0.91, 1.19) | | | | | .59 | | |
| Mean (SD) events per patient | 0.4 (1.1) | | 0.5 (1.2) | | 0.4 (0.9) | | | | | 0.08 (0.04, 0.13) | | <.001 | | | 0.01 (−0.05, 0.07) | | | | | | .77 | |
| Mean (SD) events per treated patient^c^ | 1.6 (1.6) | | 1.7 (1.8) | | 1.6 (1.1) | | | | | 0.09 (−0.01, 0.20) | | .08 | | | −0.01 (−0.16, 0.15) | | | | | | .87 | |
| **All-cause office visits** | | | | | | | | | | | | | | | | | | | | | | |
| No. (%) of patients with ≥1 event | 187,292 (93.0) | | 2,049 (90.1) | | 934 (92.8) | | | 0.68 (0.59, 0.78) | | | <.001 | | | | | 0.96 (0.76, 1.22) | | | .76 | | | |
| Mean (SD) events per patient | 10.2 (10.2) | | 10.9 (11.2) | | 12.3 (12.3) | | | 0.64 (0.22, 1.07) | | | .002 | | | | | 2.10 (1.41, 2.83) | | | 0 | | | |
| Mean (SD) events per treated patient^c^ | 11.0 (10.2) | | 12.1 (11.2) | | 13.3 (12.2) | | | 1.07 (0.65, 1.50) | | | 0 | | | | | 2.29 (1.62, 3.00) | | | 0 | | | |
| **All-cause outpatient visits** | | | | | | | | | | | | | | | | | | | | | | |
| No. (%) of patients with ≥1 event | 180,587 (89.7) | | 2,011 (88.4) | | 908 (90.2) | | | 0.88 (0.77, 1.00) | | | .05 | | | | | 1.06 (0.86, 1.30) | | | .60 | | | |
| Mean (SD) events per patient | 18.4 (28.6) | | 22.5 (34.8) | | 26.5 (36.2) | | | 4.14 (3.03, 5.31) | | | 0 | | | | | 8.12 (6.19, 10.20) | | | 0 | | | |
| Mean (SD) events per treated patient^c^ | 20.5 (29.5) | | 25.5 (36.0) | | 29.4 (37.0) | | | 4.98 (3.87, 6.15) | | | 0 | | | | | 8.89 (7.01, 10.89) | | | 0 | | | |
| **All-cause SNF visits** | | | | | | | | | | | | | | | | | | | | | | |
| No. (%) of patients with ≥1 event | 13,161 (6.5) | | 359 (15.8) | | 144 (14.3) | | | 2.68 (2.39, 3.00) | | | <.001 | | | | | 2.39 (2.00, 2.85) | | | <.001 | | | |
| Mean (SD) events per patient | 0.9 (6.6) | | 3.3 (14.3) | | 2.7 (11.8) | | | 2.37 (1.48, 3.58) | | | 0 | | | | | 1.76 (0.75, 3.38) | | | <.001 | | | |
| Mean (SD) events per treated patient^c^ | 13.9 (21.8) | | 20.8 (30.5) | | 18.7 (26.0) | | | 6.84 (4.61, 9.35) | | | 0 | | | | | 4.74 (1.69, 8.39) | | | .001 | | | |
| **Any antibiotic use** | | | | | | | | | | | | | | | | | | | | | | |
| No. (%) of patients with ≥1 event | 50,854 (25.3) | | 646 (28.4) | | 344 (34.2) | | | | 1.17 (1.07, 1.29) | | <.001 | | | | | 1.54 (1.35, 1.75) | | | <.001 | | | |
| Mean (SD) events per patient | 0.4 (0.8) | | 0.5 (1.0) | | 0.6 (1.2) | | | | 0.10 (0.06, 0.14) | | 0 | | | | | 0.24 (0.17, 0.32) | | | 0 | | | |
| Mean (SD) events per treated patient^c^ | 1.6 (1.0) | | 1.7 (1.1) | | 1.9 (1.3) | | | | 0.16 (0.06, 0.25) | | <.001 | | | | | 0.27 (0.14, 0.41) | | | <.001 | | | |
| **Post-index utilization (30 days)^d^** | | | | | | | | | | | | | | | | | | | | | | |
| No. of patients completing 30 day follow up period | n = 192,204 | | n = 1,762 | | n = 732 | | | |  | |  | | | | |  | | |  | | | |
| **All-cause ED visits** | | | | | | | | | | | | | | | | | | | | | | |
| No. (%) of patients with ≥1 event | 14,141 (7.4) | | 163 (9.3) | | 74 (10.1) | | | | 1.28 (1.09, 1.51) | | .003 | | | | | 1.42 (1.11, 1.80) | | | | .004 | | |
| Mean (SD) events per patient | 0.1 (0.3) | | 0.1 (0.4) | | 0.1 (0.3) | | | | 0.02 (0.01, 0.04) | | <.001 | | | 0.03 (0.01, 0.05) | | | | | | .01 | | |
| Mean (SD) events per treated patient^c^ | 1.1 (0.4) | | 1.2 (0.5) | | 1.1 (0.3) | | | | 0.04 (−0.12, 0.22) | | .65 | | | −0.03 (−0.25, 0.23) | | | | | | .78 | | |
| **All-cause office visits** | | | | | | | | | | | | | | | | | | | | | | |
| No. (%) of patients with ≥1 event | 143,873 (74.9) | | 1179 (66.9) | | 448 (61.2) | 0.68 (0.62, 0.75) | | | | | <.001 | | | | | 0.53 (0.46, 0.62) | | | | | | <.001 |
| Mean (SD) events per patient | 3.0 (5.1) | | 3.2 (5.9) | | 3.0 (5.7) | 0.19 (0.01, 0.38) | | | | | .04 | | | | | 0.02 (-0.24, 0.30) | | | | | | .88 |
| Mean (SD) events per treated patient^c^ | 4.0 (5.5) | | 4.8 (6.6) | | 4.9 (6.5) | 0.76 (0.52, 1.00) | | | | | <.001 | | | | | 0.93 (0.55, 1.34) | | | | | | <.001 |
| **All-cause outpatient visits** | | | | | | | | | | | | | | | | | | | | | | |
| No. (%) of patients with ≥1 event | 135,501 (70.5) | | 1,405 (79.7) | | 578 (79.0) | 1.65 (1.47, 1.85) | | | | | <.001 | | | | | 1.57 (1.31, 1.88) | | | <.001 | | | |
| Mean (SD) events per treated patient^c^ | 10.7 (15.2) | | 15.8 (19.7) | | 18.2 (24.1) | | 5.12 (4.30, 5.99) | | | | <.001 | | | | | 7.48 (6.04, 9.04) | | | <.001 | | | |
| **All-cause SNF visits** | | | | | | | | | | | | | | | | | | | | | | |
| No. (%) of patients with ≥1 event | 40,770 (21.2) | | 884 (50.2) | 396 (54.1) | | 3.74 (3.40, 4.11) | | | | | <.001 | | | | | 4.38 (3.78, 5.07) | | | <.001 | | | |
| Mean (SD) events per patient | 1.7 (5.2) | | 4.7 (7.9) | 5.1 (8.3) | | 2.98 (2.25, 3.84) | | | | | <.001 | | | | | 3.38 (2.21, 4.90) | | | <.001 | | | |
| Mean (SD) events per treated patient^c^ | 8.2 (8.7) | | 9.4 (9.0) | 9.5 (9.3) | | 1.20 (0.72, 1.69) | | | | | <.001 | | | | | 1.26 (0.56, 2.01) | | | <.001 | | | |
| **Post-index utilization (90 days)^d^** | | | | | | | | | | | | | | | | | | | | | | |
| No. of patients completing 90 day follow up period | n = 189,597 | | n = 1,685 | n = 687 | |  | | | | |  | | | | |  | |  | | | | |
| **All-cause ED visits** | | | | | | | | | | | | | | | | | | | | | | |
| No. (%) of patients with ≥1 event | 25,933 (13.7) | | 294 (17.4) | 124 (18.0) | | 1.33 (1.18, 1.51) | | | | | <.001 | | | | | 1.39 (1.15, 1.69) | | <.001 | | | | |
| Mean (SD) events per patient | 0.2 (0.5) | | 0.2 (0.6) | 0.2 (0.6) | | 0.07 (0.04, 0.09) | | | | | 0 | | | | | 0.06 (0.03, 0.10) | | <.001 | | | | |
| Mean (SD) events per treated patient^c^ | 1.3 (0.7) | | 1.4 (0.8) | 1.3 (0.7) | | 0.10 (-0.03, 0.24) | | | | | .12 | | | | | 0.03 (-0.15, 0.25) | | .75 | | | | |
| **All-cause office visits** | | | | | | | | | | | | | | | | | | | | | | |
| No. (%) of patients with ≥1 event | 171,981 (90.7) | | 1,411 (83.7) | | 555 (80.8) | | 0.53 (0.46, 0.60) | | | | <.001 | | 0.43 (0.36, 0.52) | | | | | | | | <.001 | |
| Mean (SD) events per patient | 7.6 (11.9) | | 8.7 (15.2) | | 8.3 (13.2) | | 1.08 (0.65, 1.53) | | | | <.001 | | 0.71 (0.07, 1.40) | | | | | | | | .03 | |
| Mean (SD) events per treated patient^c^ | 8.4 (12.3) | | 10.4 (16.0) | | 10.3 (14.0) | | 1.99 (1.50, 2.49) | | | | <.001 | | 1.91 (1.16, 2.72) | | | | | | | | <.001 | |
| **All-cause outpatient visits** | | | | | | | | | | | | | | | | | | | | | | |
| No. (%) of patients with ≥1 event | 166,271 (87.7) | | 1,542 (91.5) | | 628 (91.4) | | 1.51 (1.27, 1.79) | | | | <.001 | | 1.49 (1.14, 1.95) | | | | | | | | .003 | |
| Mean (SD) events per patient | 21.7 (34.2) | | 33.0 (43.8) | | 39.5 (54.4) | | 11.26 (9.25, 13.41) | | | | <.001 | | 17.80 (14.09, 21.89) | | | | | | | | <.001 | |
| Mean (SD) events per treated patient^c^ | 24.7 (35.4) | | 36.0 (44.6) | | 43.2 (55.5) | | 11.28 (9.41, 13.25) | | | | <.001 | | 18.47 (15.02, 22.21) | | | | | | | | <.001 | |
| **All-cause SNF visits** | | | | | | | | | | | | | | | | | | | | | | |
| No. (%) of patients with ≥1 event | 42,448 (22.4) | | 868 (51.5) | | 394 (57.4) | | 3.68 (3.35, 4.06) | | | | <.001 | | 4.66 (4.01, 5.42) | | | | | | | | <.001 | |
| Mean (SD) events per patient | 3.0 (9.9) | | 9.1 (16.5) | | 10.4 (16.7) | | 6.17 (4.66, 7.99) | | | | <.001 | | 7.48 (4.90, 10.90) | | | | | | | | <.001 | |
| Mean (SD) events per treated patient^c^ | 13.2 (17.4) | | 17.7 (19.4) | | 18.2 (18.6) | | 4.55 (3.48, 5.69) | | | | <.001 | | 5.02 (3.43, 6.77) | | | | | | | | <.001 | |

CI=confidence interval; ED=emergency department; ICU=intensive care unit; SD=standard deviation; SNF=skilled nursing facility.

^a^Odds ratio (OR) from Chi-square test is used for categorical variables, negative binomial models are used to test for differences between means for count variables (number of events and length of stay). Statistical comparisons are comparing Cohort 3 (pneumonia due to *S. aureus*) with Cohort 1 (no pneumonia, reference group); ie, Difference = mean (Cohort 3) – mean (Cohort 1) and OR = Odds (Cohort 3)/Odds (Cohort 1).

^b^Odds ratio (OR) from Chi-square test is used for categorical variables, negative binomial models are used to test for differences between means for count variables (number of events and length of stay). Statistical comparisons are comparing Cohort 4 (pneumonia due to *P. aeruginosa*) with Cohort 1 (no pneumonia, reference group); ie, Difference = mean (Cohort 4) – mean (Cohort 1) and OR = Odds (Cohort 4)/Odds (Cohort 1).

^c^Including only patients with at least one event.

^d^Post-index utilization results include only patients who survived through the end of the 90 day follow up period.

eTable 4. Comorbidities of ICU patients with *S. aureus* and *P.* *aeruginosa* pneumonia versus controls

|  | **Cohorts of Interest** | | | **Comparisons** | | | | |
| --- | --- | --- | --- | --- | --- | --- | --- | --- |
|  | **No pneumonia, mean (SD) (n=201,394)** | ***S. aureus* pneumonia, mean (SD) (n=2,275)** | ***P. aeruginosa* pneumonia, mean (SD) (n=1,007)** | ***S. aureus* pneumonia vs No pneumonia** | | | ***P. aeruginosa* pneumonia vs No pneumonia** | |
|  |  |  |  | **Difference^a^ (95% CI)** | ***P* value^a^** | | **Difference^b^ (95% CI)** | ***P* value^b^** |
| **No. (%) of patients with comorbidity** | | | | | | | | |
| Diabetes | 49,321 (24.5) | 664 (29.2) | 267 (26.5) | 1.27 (1.16, 1.39) | <.001 | 1.11 (0.97, 1.28) | | .14 |
| Myocardial infarction | 11,970 (5.9) | 136 (6.0) | 67 (6.7) | 1.01 (0.85, 1.20) | .94 | 1.13 (0.88, 1.45) | | .34 |
| Congestive heart failure | 22,939 (11.4) | 431 (18.9) | 204 (20.3) | 1.82 (1.64, 2.02) | <.001 | 1.98 (1.69, 2.31) | | <.001 |
| Peripheral artery disease | 20,880 (10.4) | 279 (12.3) | 138 (13.7) | 1.21 (1.07, 1.37) | .003 | 1.37 (1.15, 1.65) | | <.001 |
| Stroke /TIA / Cerebrovascular disease | 30,704 (15.2) | 368 (16.2) | 143 (14.2) | 1.07 (0.96, 1.20) | .22 | 0.92 (0.77, 1.10) | | .36 |
| Hypertension | 113,336 (56.3) | 1,321 (58.1) | 618 (61.4) | 1.08 (0.99, 1.17) | .09 | 1.23 (1.09, 1.40) | | .001 |
| Coronary heart disease | 55,658 (27.6) | 561 (24.7) | 279 (27.7) | 0.86 (0.78, 0.94) | .002 | 1.00 (0.87, 1.15) | | .96 |
| Anemia | 35,063 (17.4) | 566 (24.9) | 276 (27.4) | 1.57 (1.43, 1.73) | <.001 | 1.79 (1.56, 2.06) | | <.001 |
| COPD | 26,476 (13.1) | 617 (27.1) | 359 (35.7) | 2.46 (2.24, 2.70) | <.001 | 3.66 (3.22, 4.17) | | <.001 |
| Asthma | 16,187 (8.0) | 290 (12.7) | 146 (14.5) | 1.67 (1.48, 1.89) | <.001 | 1.94 (1.63, 2.31) | | <.001 |
| Renal disease | 16,382 (8.1) | 264 (11.6) | 134 (13.3) | 1.48 (1.30, 1.69) | <.001 | 1.73 (1.44, 2.08) | | <.001 |
| Chronic liver disease | 12,125 (6.0) | 157 (6.9) | 77 (7.6) | 1.16 (0.98, 1.36) | .08 | 1.29 (1.02, 1.63) | | .03 |
| Neutropenia | 2,795 (1.4) | 53 (2.3) | 31 (3.1) | 1.70 (1.29, 2.23) | <.001 | 2.26 (1.58, 3.24) | | <.001 |
| Immunosuppression | 1,096 (0.5) | 26 (1.1) | 19 (1.9) | 2.12 (1.43, 3.13) | <.001 | 3.52 (2.22, 5.56) | | <.001 |
| HIV | 459 (0.2) | 11 (0.5) | 9 (0.9) | 2.13 (1.17, 3.88) | .01 | 3.95 (2.04, 7.66) | | <.001 |
| Hepatitis B | 424 (0.2) | 6 (0.3) | 4 (0.4) | 1.25 (0.56, 2.81) | .49 | 1.89 (0.71, 5.07) | | .17 |
| Hepatitis C | 1,570 (0.8) | 22 (1.0) | 11 (1.1) | 1.24 (0.82, 1.90) | .31 | 1.41 (0.78, 2.56) | | .26 |
| Obesity/Overweight | 15,163 (7.5) | 182 (8.0) | 82 (8.1) | 1.07 (0.92, 1.24) | .40 | 1.09 (0.87, 1.37) | | .46 |
| Dementia | 2,313 (1.1) | 78 (3.4) | 18 (1.8) | 3.06 (2.43, 3.85) | <.001 | 1.57 (0.98, 2.50) | | .06 |
| Dialysis | 2,748 (1.4) | 45 (2.0) | 23 (2.3) | 1.46 (1.08, 1.96) | .01 | 1.69 (1.12, 2.56) | | .01 |
| Leukemia | 1,321 (0.7) | 21 (0.9) | 20 (2.0) | 1.41 (0.92, 2.18) | .12 | 3.07 (1.97, 4.79) | | <.001 |
| Lymphoma | 2,251 (1.1) | 42 (1.8) | 25 (2.5) | 1.66 (1.22, 2.26) | .001 | 2.25 (1.51, 3.36) | | <.001 |
| Cancer other than leukemia or lymphoma | 4,876 (2.4) | 78 (3.4) | 41 (4.1) | 1.43 (1.14, 1.80) | .002 | 1.71 (1.25, 2.34) | | <.001 |
| Bone marrow transplant | 234 (0.1) | 4 (0.2) | 5 (0.5) | 1.51 (0.56, 4.07) | .35 | 4.29 (1.77, 10.43) | | .007 |
| Solid organ transplant | 1,714 (0.9) | 35 (1.5) | 31 (3.1) | 1.82 (1.30, 2.55) | <.001 | 3.70 (2.58, 5.31) | | <.001 |
| **No. (%) of patients with Deyo Charlson comorbidity score (DCI)** | | | | | | | | |
| 0 | 80,861 (40.2) | 736 (32.4) | 279 (27.7) | 0.71 (0.65, 0.78) | <.001 | | 0.57 (0.50, 0.66) | <.001 |
| 1–2 | 70,066 (34.8) | 768 (33.8) | 335 (33.3) | 0.96 (0.88, 1.04) | .30 | | 0.94 (0.82, 1.07) | .31 |
| 3–4 | 28,816 (14.3) | 375 (16.5) | 196 (19.5) | 1.18 (1.06, 1.32) | .003 | | 1.45 (1.24, 1.69) | <.001 |
| 5+ | 21,651 (10.8) | 396 (17.4) | 197 (19.6) | 1.75 (1.57, 1.95) | <.001 | | 2.02 (1.73, 2.36) | <.001 |
| Mean (SD) DCI score | 1.7 (2.3) | 2.3 (2.8) | 2.5 (2.8) | 0.57 (0.48, 0.67) | <.001 | | 0.80 (0.65, 0.94) | <.001 |

CI=confidence interval; COPD=chronic obstructive pulmonary disease; HIV=human immunodecifiency virus; ICU=intensive care unit; SD=standard deviation; TIA=transient ischemic attack.

^a^Odds ratio (OR) from chi-square test is used for categorical variables. Statistical comparisons are comparing Cohort 3 (pneumonia due to *S. aureus*) with Cohort 1 (no pneumonia, reference group); ie, OR = Odds (Cohort 3)/Odds (Cohort 1).

^b^Odds ratio (OR) from chi-square test is used for categorical variables. Statistical comparisons are comparing Cohort 4 (pneumonia due to *P. aeruginosa*) with Cohort 1 (no pneumonia, reference group); ie, OR = Odds (Cohort 4)/Odds (Cohort 1).

eTable 5. Prevalence of comorbid conditions by quintile of index hospitalization cost for ICU patients with *S. aureus* or *P. aeruginosa* pneumonia versus controls

|  | **1st Quintile** | **2nd Quintile** | **3rd Quintile** | **4th Quintile** | **5th Quintile** |
| --- | --- | --- | --- | --- | --- |
| **No pneumonia** |  |  |  |  |  |
| Cost range, $ | 0–4,130 | 4,130–13,209 | 13,209–25,719 | 25,719–50,643 | 50,643–2,122,955 |
| No. of patients | 40,279 | 40,279 | 40,279 | 40,279 | 40,278 |
| Comorbidity, n (%) |  |  |  |  |  |
| Diabetes | 12,493 (31.0) | 10,146 (25.2) | 9,524 (23.6) | 8,710 (21.6) | 8,448 (21.0) |
| MI | 3,525 (8.8) | 2,056 (5.1) | 2,153 (5.3) | 2,232 (5.5) | 2,004 (5.0) |
| CHF | 8,457 (21.0) | 3,969 (9.9) | 3,719 (9.2) | 3,175 (7.9) | 3,619 (9.0) |
| Peripheral artery disease | 7,413 (18.4) | 3,476 (8.6) | 3,813 (9.5) | 3,474 (8.6) | 2,704 (6.7) |
| Stroke, TIA, cerebrovascular disease | 10,045 (24.9) | 5,644 (14.0) | 5,242 (13.0) | 5,232 (13.0) | 4,541 (11.3) |
| Hypertension | 30,538 (75.8) | 20,858 (51.8) | 21,153 (52.5) | 20,947 (52.0) | 19,840 (49.3) |
| Other coronary heart disease | 16,868 (41.9) | 8,482 (21.1) | 9,173 (22.8) | 10,488 (26.0) | 10,647 (26.4) |
| Anemia | 9,622 (23.9) | 6,507 (16.2) | 6,670 (16.6) | 6,086 (15.1) | 6,178 (15.3) |
| COPD | 8,684 (21.6) | 4,834 (12.0) | 5,030 (12.5) | 4,482 (11.1) | 3,446 (8.6) |
| Asthma | 3,244 (8.1) | 3,627 (9.0) | 3,357 (8.3) | 3,053 (7.6) | 2,906 (7.2) |
| Renal disease | 6,044 (15.0) | 2,908 (7.2) | 2,860 (7.1) | 2,211 (5.5) | 2,359 (5.9) |
| Chronic liver disease | 1,997 (5.0) | 2,038 (5.1) | 2,640 (6.6) | 2,627 (6.5) | 2,823 (7.0) |
| Neutropenia | 410 (1.0) | 443 (1.1) | 542 (1.3) | 626 (1.6) | 774 (1.9) |
| Immunosuppression | 165 (0.4) | 207 (0.5) | 221 (0.5) | 202 (0.5) | 301 (0.7) |
| HIV | 38 (0.1) | 74 (0.2) | 107 (0.3) | 102 (0.3) | 138 (0.3) |
| Hepatitis B | 44 (0.1) | 58 (0.1) | 89 (0.2) | 91 (0.2) | 142 (0.4) |
| Hepatitis C | 148 (0.4) | 296 (0.7) | 364 (0.9) | 321 (0.8) | 441 (1.1) |
| Obesity/Overweight | 2,274 (5.6) | 3,096 (7.7) | 3,265 (8.1) | 3,234 (8.0) | 3,294 (8.2) |
| Dementia | 1,179 (2.9) | 451 (1.1) | 394 (1.0) | 212 (0.5) | 77 (0.2) |
| Dialysis | 1,243 (3.1) | 368 (0.9) | 363 (0.9) | 287 (0.7) | 487 (1.2) |
| Leukemia | 337 (0.8) | 180 (0.4) | 228 (0.6) | 237 (0.6) | 339 (0.8) |
| Lymphoma | 555 (1.4) | 329 (0.8) | 390 (1.0) | 455 (1.1) | 522 (1.3) |
| Cancer other than leukemia or lymphoma | 1,369 (3.4) | 639 (1.6) | 870 (2.2) | 1,035 (2.6) | 963 (2.4) |
| Bone marrow transplant | 24 (0.1) | 20 (0.0) | 44 (0.1) | 33 (0.1) | 113 (0.3) |
| Solid organ transplant | 408 (1.0) | 249 (0.6) | 256 (0.6) | 251 (0.6) | 550 (1.4) |
| **Pneumonia due to *S. aureus*** | |  |  |  |  |
| Cost range, $ | 0–9,252 | 9,252–42,482 | 42,482–105,668 | 105,66–242,198 | 242,198–4,067,031 |
| No. of patients | 455 | 455 | 455 | 455 | 455 |
| Comorbidity, n (%) |  |  |  |  |  |
| Diabetes | 159 (34.9) | 143 (31.4) | 139 (30.5) | 119 (26.2) | 104 (22.9) |
| Myocardial infarction (MI) | 52 (11.4) | 33 (7.3) | 24 (5.3) | 13 (2.9) | 14 (3.1) |
| Congestive heart failure (CHF) | 135 (29.7) | 120 (26.4) | 85 (18.7) | 49 (10.8) | 42 (9.2) |
| Peripheral artery disease | 100 (22.0) | 75 (16.5) | 50 (11.0) | 32 (7.0) | 22 (4.8) |
| Stroke, TIA, cerebrovascular disease | 120 (26.4) | 92 (20.2) | 68 (14.9) | 47 (10.3) | 41 (9.0) |
| Hypertension | 344 (75.6) | 298 (65.5) | 272 (59.8) | 217 (47.7) | 190 (41.8) |
| Other coronary heart disease | 191 (42.0) | 121 (26.6) | 105 (23.1) | 82 (18.0) | 62 (13.6) |
| Anemia | 150 (33.0) | 129 (28.4) | 111 (24.4) | 96 (21.1) | 80 (17.6) |
| COPD | 187 (41.1) | 155 (34.1) | 123 (27.0) | 94 (20.7) | 58 (12.7) |
| Asthma | 58 (12.7) | 65 (14.3) | 68 (14.9) | 51 (11.2) | 48 (10.5) |
| Renal disease | 81 (17.8) | 57 (12.5) | 53 (11.6) | 38 (8.4) | 35 (7.7) |
| Chronic liver disease | 23 (5.1) | 35 (7.7) | 31 (6.8) | 35 (7.7) | 33 (7.3) |
| Neutropenia | 9 (2.0) | 9 (2.0) | 10 (2.2) | 13 (2.9) | 12 (2.6) |
| Immunosuppression | 3 (0.7) | 7 (1.5) | 4 (0.9) | 9 (2.0) | 3 (0.7) |
| HIV | 0 (0.0) | 2 (0.4) | 3 (0.7) | 4 (0.9) | 2 (0.4) |
| Hepatitis B | 0 (0.0) | 1 (0.2) | 2 (0.4) | 0 (0.0) | 3 (0.7) |
| Hepatitis C | 1 (0.2) | 7 (1.5) | 4 (0.9) | 4 (0.9) | 6 (1.3) |
| Obesity/overweight | 33 (7.3) | 38 (8.4) | 36 (7.9) | 43 (9.5) | 32 (7.0) |
| Dementia | 37 (8.1) | 21 (4.6) | 9 (2.0) | 9 (2.0) | 2 (0.4) |
| Dialysis | 18 (4.0) | 11 (2.4) | 6 (1.3) | 5 (1.1) | 5 (1.1) |
| Leukemia | 6 (1.3) | 5 (1.1) | 4 (0.9) | 5 (1.1) | 1 (0.2) |
| Lymphoma | 7 (1.5) | 7 (1.5) | 8 (1.8) | 11 (2.4) | 9 (2.0) |
| Cancer other than leukemia or lymphoma | 16 (3.5) | 26 (5.7) | 12 (2.6) | 16 (3.5) | 8 (1.8) |
| Bone marrow transplant | 0 (0.0) | 1 (0.2) | 0 (0.0) | 3 (0.7) | 0 (0.0) |
| Solid organ transplant | 9 (2.0) | 4 (0.9) | 4 (0.9) | 7 (1.5) | 11 (2.4) |
| **Pneumonia due to *P. aeruginosa*** | |  |  |  |  |
| Cost range, $ | 0–11,577 | 2,598–49,956 | 14,369–165,122 | 30,976–348,862 | 72,688–3,649,184 |
| No. of patients | 202 | 201 | 202 | 201 | 201 |
| Comorbidity, n (%) |  |  |  |  |  |
| Diabetes | 62 (30.7) | 59 (29.4) | 49 (24.3) | 58 (28.9) | 39 (19.4) |
| Myocardial infarction | 15 (7.4) | 17 (8.5) | 13 (6.4) | 11 (5.5) | 11 (5.5) |
| Congestive heart failure | 64 (31.7) | 53 (26.4) | 37 (18.3) | 30 (14.9) | 20 (10.0) |
| Peripheral artery disease | 49 (24.3) | 36 (17.9) | 27 (13.4) | 18 (9.0) | 8 (4.0) |
| Stroke, TIA, cerebrovascular disease | 37 (18.3) | 35 (17.4) | 30 (14.9) | 23 (11.4) | 18 (9.0) |
| Hypertension | 147 (72.8) | 141 (70.1) | 135 (66.8) | 106 (52.7) | 89 (44.3) |
| Other coronary heart disease | 77 (38.1) | 68 (33.8) | 58 (28.7) | 44 (21.9) | 32 (15.9) |
| Anemia | 61 (30.2) | 58 (28.9) | 58 (28.7) | 55 (27.4) | 44 (21.9) |
| COPD | 104 (51.5) | 108 (53.7) | 68 (33.7) | 48 (23.9) | 31 (15.4) |
| Asthma | 43 (21.3) | 31 (15.4) | 30 (14.9) | 19 (9.5) | 23 (11.4) |
| Renal disease | 37 (18.3) | 32 (15.9) | 22 (10.9) | 24 (11.9) | 19 (9.5) |
| Chronic liver disease | 15 (7.4) | 16 (8.0) | 16 (7.9) | 15 (7.5) | 15 (7.5) |
| Neutropenia | 5 (2.5) | 8 (4.0) | 9 (4.5) | 5 (2.5) | 4 (2.0) |
| Immunosuppression | 6 (3.0) | 2 (1.0) | 4 (2.0) | 4 (2.0) | 3 (1.5) |
| HIV | 2 (1.0) | 3 (1.5) | 2 (1.0) | 1 (0.5) | 1 (0.5) |
| Hepatitis B | 3 (1.5) | 1 (0.5) | 0 (0.0) | 0 (0.0) | 0 (0.0) |
| Hepatitis C | 2 (1.0) | 0 (0.0) | 4 (2.0) | 4 (2.0) | 1 (0.5) |
| Obesity/overweight | 13 (6.4) | 16 (8.0) | 16 (7.9) | 25 (12.4) | 12 (6.0) |
| Dementia | 2 (1.0) | 8 (4.0) | 3 (1.5) | 4 (2.0) | 1 (0.5) |
| Dialysis | 5 (2.5) | 8 (4.0) | 2 (1.0) | 4 (2.0) | 4 (2.0) |
| Leukemia | 3 (1.5) | 6 (3.0) | 4 (2.0) | 4 (2.0) | 3 (1.5) |
| Lymphoma | 4 (2.0) | 4 (2.0) | 9 (4.5) | 5 (2.5) | 3 (1.5) |
| Cancer other than leukemia or lymphoma | 10 (5.0) | 8 (4.0) | 9 (4.5) | 9 (4.5) | 5 (2.5) |
| Bone marrow transplant | 0 (0.0) | 2 (1.0) | 1 (0.5) | 1 (0.5) | 1 (0.5) |
| Solid organ transplant | 4 (2.0) | 5 (2.5) | 5 (2.5) | 10 (5.0) | 7 (3.5) |

CHF=congestive heart failure; COPD=chronic obstructive pulmonary disease; HIV=human immunodeficiency virus; MI=myocardial infarction; TIA=transient ischemic attack.

eTable 6. Exploratory analysis of the impact of congestive heart failure on index hospitalization costs in ICU patients with hospital acquired pneumonia due to *S. aureus*

| **Model** | **1st Quintile^a^ $0–$9,252, n/N (%)** | **2nd Quintile^a^ $9,252–$42,482, n/N (%)** | **3rd Quintile^a^ $42,482–$105,668, n/N (%)** | **4th Quintile^a^ $105,668–$242,198, n/N (%)** | **5th Quintile^a^ $242,198–$4,067,031, n/N (%)** | **Odds Ratio^b^ (95% CI)** | ***P* Value^b^** |
| --- | --- | --- | --- | --- | --- | --- | --- |
| Unadjusted | 135/455 (29.7) | 120/455 (26.4) | 85/455 (18.7) | 49/455 (10.8) | 42/455 (9.2) | 0.40 (0.33, 0.48) | <.001 |
| Adjusting for age only | 135/455 (29.7) | 120/455 (26.4) | 85/455 (18.7) | 49/455 (10.8) | 42/455 (9.2) | 0.78 (0.64, 0.95) | .014 |
| Adjusted for all covariates | 135/455 (29.7) | 120/455 (26.4) | 85/455 (18.7) | 49/455 (10.8) | 42/455 (9.2) | 0.74 (0.60, 0.92) | .007 |
| Patients ≥65 only | 127/397 (32.0) | 92/234 (39.3) | 58/167 (34.7) | 24/102 (23.5) | 14/63 (22.2) | 0.81 (0.60, 1.08) | .15 |
| Patients <65 only | 8/58 (13.8) | 28/221 (12.7) | 27/288 (9.4) | 25/353 (7.1) | 28/392 (7.1) | 0.78 (0.55, 1.12) | .18 |
| Patients not dying during index hospitalization | 101/360 (28.1) | 87/381 (22.8) | 63/378 (16.7) | 42/388 (10.8) | 34/405 (8.4) | 0.77 (0.60, 0.98) | .03 |
| Patients dying during index hospitalization | 34/95 (35.8) | 33/74 (44.6) | 22/77 (28.6) | 7/67 (10.4) | 8/50 (16.0) | 0.61 (0.38, 0.98) | .04 |
| Patients ≥65 and not dying during index hospitalization | 96/313 (30.7) | 65/179 (36.3) | 41/122 (33.6) | 20/75 (26.7) | 12/54 (22.2) | 0.91 (0.65, 1.29) | .60 |

CI=confidence interval; ICU=intensive care unit.

^a^Values reported for each cost quintile represent the number of patients with congestive heart failure in the quintile (n) divided by the total number of patients in the quintile (N).

^b^Ordinal logistic regression odds ratio (OR) is used to test the association between the presence of each comorbidity and index hospitalization cost quintiles, ie, OR=Odds (patient with comorbidity in high cost quintile)/Odds (patient without comorbidity in high cost quintile). OR >1 means presence of the comorbidity leads to greater costs, whereas OR <1 means presence of the comorbidity leads to lower costs.

All statistical models were controlled for the following variables: age (continuously), gender, health plan type, geographic region, prior inpatient hospitalization costs during the 12 month pre-index period, prior healthcare utilization during the 12 month pre-index period (inpatient stays [0 vs 1+], emergency room visits [0 vs 1+], outpatient and office visits [continuous]), and prior antibiotic use during the 12 month pre-index period (0 vs. 1+).

**eTable 7. Healthcare resource utilization for ICU patients with *S. aureus* or *P. aeruginosa* pneumonia versus controls pre-index hospitalization (12 months)**

|  | **Cohorts of interest** | | | ***S. aureus* pneumonia  vs No pneumonia** | | ***P. aeruginosa* pneumonia  vs No pneumonia** | |
| --- | --- | --- | --- | --- | --- | --- | --- |
|  | **No pneumonia**  **n=201,394** | ***S. aureus* pneumonia**  **N=2275** | ***P. aeruginosa* pneumonia**  **N=1007** | **Odds Ratio / Difference^a^ (95% CI)** | ***P* Value^a^** | **Odds Ratio / Difference^b^ (95% CI)** | ***P* Value^b^** |
| All-cause inpatient hospitalizations |  |  |  |  |  |  |  |
| No. (%) of patients with ≥1 event | 59,926 (29.8) | 853 (37.5) | 409 (40.6) | 1.42 (1.30, 1.54) | <.001 | 1.62 (1.42, 1.83) | <.001 |
| Mean (SD) events per patient with at least 2 events^c^ | 1.7 (1.5) | 2.2 (2.1) | 2.1 (1.9) | 0.43 (0.33, 0.54) | 0 | 0.41 (0.26, 0.56) | 0 |
| Mean (SD) length of stay per patient with at least 1 event^c^ | 7.4 (12.4) | 13.4 (20.3) | 14.6 (24.6) | 6.10 (5.15, 7.11) | 0 | 7.21 (5.77, 8.82) | 0 |
